# pH-Tolerant giant vesicles composed of cationic lipids with imine linkages and oleic acids†

**DOI:** 10.1039/d0ra06822e

**Published:** 2020-09-15

**Authors:** Daichi Sawada, Ayana Hirono, Kouichi Asakura, Taisuke Banno

**Affiliations:** a Department of Applied Chemistry, Faculty of Science and Technology, Keio University 3-14-1 Hiyoshi, Kohoku-ku Yokohama 223-8522 Japan banno@applc.keio.ac.jp

## Abstract

Giant vesicles (GVs) have attracted attention as functional materials because they can encapsulate both hydrophilic and hydrophobic compounds. For next generation functional GVs, both tolerance and stimuli-sensitivity are needed. So far, vesicles tolerant to acidic or basic conditions were generated using a mixture of cationic lipids and fatty acids. Here, to create functional GVs that are tolerant to a wide pH range but sensitively respond at below a specific pH, the behaviour of GVs composed of a cationic lipid with an imine bond and oleic acid was investigated. Even though the GVs prepared by the film swelling method were tolerant to strongly acidic conditions, GVs without oleic acid gradually shrank, accompanied by the generation of oil droplets at the same pH. ^1^H NMR analysis revealed that during hydration of the film, the imine bond hydrolysed to provide a cationic surfactant and an oil component in the presence of oleic acid due to its own Lewis basicity, suggesting the dissociation of oleic acid. The results of fluorescence spectroscopy using an environment-responsive probe and IR spectroscopy indicated that the GV tolerance originated from the intermolecular interactions of cationic lipids and anionic oleate.

## Abstract 2

Giant vesicles composed of cationic lipids having an imine linkage and oleic acid were stable at strong acidic conditions.

## Graphical abstract

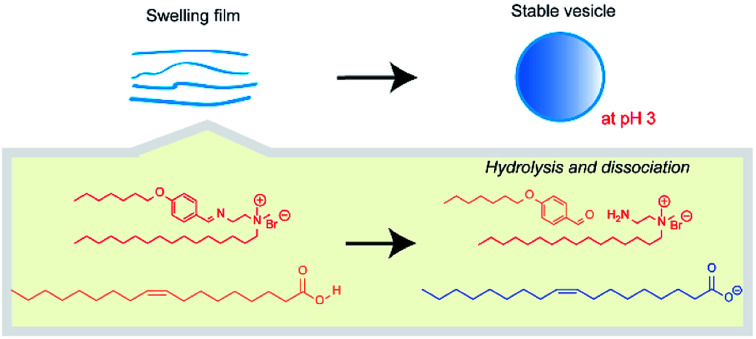

## Introduction

Amphiphilic molecules form various molecular aggregates in water depending on their hydrophilicity and hydrophobicity. Among them, vesicles in which the bilayer membranes are closed like a bag can encapsulate both hydrophilic and hydrophobic compounds because of their characteristic structure. Directed toward functional materials, many stimuli-responsive vesicles were reported using precisely designed amphiphilic molecules. For example, the introduction of transmembrane protein-like functional molecules into the vesicular membrane provides notable properties, such as releasing^1–5^ and uptaking compounds from the external and internal phases, respectively, under specific conditions.^6–8^ Vesicles are also useful as micro reaction fields for analysis in biological systems and as tissue markers.^9–12^ In addition, it is well known that polymersomes of amphipathic polymers are more stable than vesicles composed of low-molecular-weight amphiphiles under various conditions. The shape of nanometer-sized polymersomes differs according to the composition of the polymer chain, and the affinity with cells depends on their shape; therefore, polymersomes are useful for nanomedicines.^13–15^ The structural durability of vesicles is one of the important factors for functional material applications. Although polymersomes are generally stable, their responsiveness to stimuli is relatively slow. Moreover, vesicles composed of low-molecular-weight amphiphiles sensitively respond to stimuli. However, they are fragile and rapidly collapse even under relatively mild conditions.

To resolve such a dilemma between stability and stimuli-responsiveness, functional vesicles composed of mixtures of cationic and anionic surfactants and other compounds were recently reported.^16–19^ They exhibit stable macroscopic structures but the size decreases in response to stimuli, such as ion addition, pH change, and temperature. In addition, it is known that anionic surfactants self-assemble in the presence of cationic surfactants to form a unique molecular assembly.^20,21^ Fatty acids are also key compounds creating stable vesicles. Suga *et al.* reported that, although oleic acid forms vesicles at a pH of 8.0–9.8, the stable pH range extended to 10.6 by adding dodecyldimethylammonium bromide.^22^ However, the creation of tolerant vesicles under both acidic and basic conditions is still challenging.

**Fig. 1:**
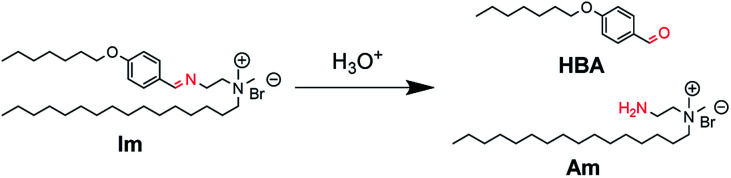
Hydrolysis of Im to generate oil component HBA and cationic surfactant Am under acidic conditions.

Herein, we report catanionic giant vesicles (GVs) that are tolerant to a wide pH range but fragile, rapidly collapsing at a specific pH. We have previously reported that the morphology of GVs composed of amphiphiles having polymerisable and hydrolysable groups depends on the lipid composition, including polymers and hydrolysates.^23,24^ These results suggest that the membrane structures that induce a drastic change in GVs are affected by molecular packing based on intermolecular interactions between lipid building blocks. Thus, a cationic lipid having an imine bond (Im), which is hydrolysed under acidic conditions to produce the oil component HBA and the cationic surfactant Am, was designed (Fig. 1). By coexisting Im with oleic acid, which affords vesicles under weak basic conditions,^25,26^ not only the hydrolysis ratio of Im but also the acid dissociation degree of oleic acid could change depending on pH.^27–29^ GVs that are tolerant from basic to acidic conditions but rapidly collapse below a certain acidic pH are created because of the different molecular packing of the vesicular membrane.

## Materials and methods

### General

Commercial reagents and solvents were purchased from Tokyo Chemical Industry Co., Ltd. (Tokyo, Japan), Wako Chemical Industry Co., Ltd. (Osaka, Japan), and Kanto Chemical Co., Ltd. (Tokyo, Japan). They were used without further purification. Synthetic procedures for the compounds are described in the ESI.† The compounds were identified by ^1^H NMR and mass spectrometry. The ^1^H NMR apparatus was an ECA-400 Fourier transform spectrometer (JEOL Ltd., Tokyo, Japan), and the chemical shift was based on tetramethylsilane (0 ppm). Mass spectrometry was performed by electrospray ionisation using an LCT-Premier (Waters Corp., Milford, MA, USA).

## Methods

### Preparation of GVs by thin-film swelling method and microscopic observation

Aliquots of 20 mM Im and 20 mM oleic acid chloroform solutions were prepared at room temperature (23–25 °C). A 2 mM lipid solution (2 mL) was formulated and dried under reduced pressure for 2 h or more to form a thin film. A volume of 4 millilitres of distilled water was gently added to swell the film at room temperature (23–25 °C) for 2 h. The final lipid concentration was 1 mM of Im/oleic acid = 100/0, 75/25, 50/50, 25/75, and 0/100 (mol%). After stirring for 10 s using a vortex mixer (TM-1, AS ONE, Osaka), the desired amount of 1 M HCl or 1 M NaOH solution was added to the sample dispersion to adjust the pH. Then, the sample (75 μL) was sealed in a glass chamber (15 × 15 × 0.28 mm; frame seal chamber, MJ Research Inc., Waltham) and observed under an optical microscope (BX51, Olympus, Tokyo, Japan) equipped with a CCD camera (DP22, Olympus, Tokyo, Japan). Fluorescence microscopy observations of GVs containing Texas Red-DHPE, uranine, or Laurdan were captured using a mercury lamp excitation source and filters for excitation and emission (*λ*_ex_ = 473 nm and *λ*_em_ = 490–590 nm for Texas Red-DHPE, *λ*_ex_ = 592 nm and *λ*_em_ = 612 nm for uranine, and *λ*_ex_ = 359 nm and *λ*_em_ = 461 nm for Laurdan) under a confocal laser microscope (FV10i-DOC, Olympus). The concentration of all fluorescent dyes was 1 mM.

### Time course measurement of the Im hydrolysis

A 7.5 mM dispersion of Im or 10 mM of Im/oleic acid = 75/25 (mol%) was prepared similar to the procedure for microscopy observations using D_2_O. To adjust the pH of the dispersions, HCl or NaOH aqueous solution was added to be 10^−4^ to 10^−2^ M. The total volume of each sample dispersion was 100 μL. After the samples were kept at room temperature (23–25 °C) for 24 h, 650 μL of CD_3_OD-*d*_4_ containing DMF (36.5 mM) was added as a standard compound to homogenise the samples. The molecular conversion of Im was analysed by ^1^H NMR spectroscopy. Hydrolytic degradation of the imine was calculated by integrating the imine proton peak at *δ* 8.38 ppm of Im and the aldehyde proton at *δ* 9.78 ppm of HBA. The measurements were performed in triplicate.

### Fluorescence emission

Initially, 20 mM and 100 mM chloroform solutions of lipids and Laurdan, respectively, were prepared. A mixture of 200 μL of lipid solution, 40 μL of Laurdan solution, and 1.8 mL of chloroform was dried under reduced pressure at room temperature (23–25 °C) for 2 h or more to form thin films. Four millilitres of distilled water was gently added to swell the film at room temperature for 2 h. The final lipid and Laurdan concentrations were 1 mM and 1 μM, respectively. Then, the samples were mixed using a vortex mixer for 10 s, and the fluorescence spectra of Laurdan were recorded using a fluorometer (RF-6000, Shimadzu Co., Ltd., Kyoto, Japan) in the fluorescence wavelength range of 380 nm to 600 nm, while the excitation wavelength was set at 340 nm. The spectra of the vesicle dispersion containing hydrolysates of Im and 1,6-diphenyl-1,3,5-hexatriene (DPH) were also recorded by setting excitation and emission at 360 and 430 nm, respectively.

## Results and discussion

### Microscopic observations of GVs under various pH conditions

**Fig. 2:**
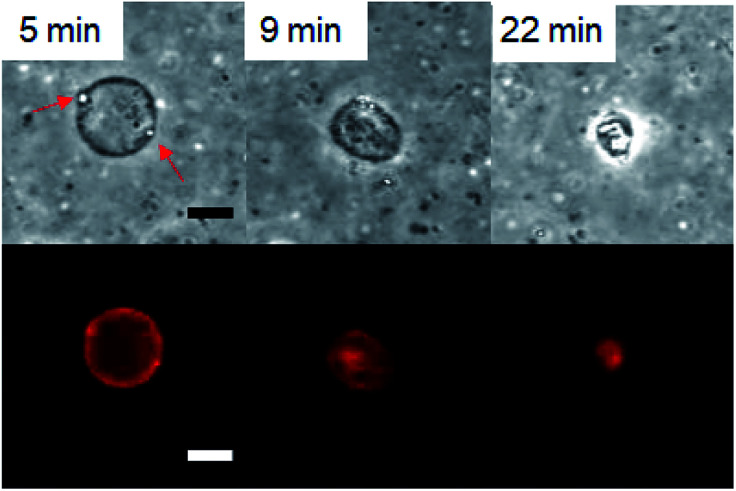
Confocal laser microscope bright field (top) and fluorescence (bottom) images of shrinkage of GVs composed of Im. Droplets indicated by red arrows generated at the membrane. Scale bar: 10 μm.

First, the GVs composed of hydrolysable Im that were prepared according to a thin film swelling method were observed under various pH conditions at room temperature (23–25 °C). Using either a fluorescent lipid, Texas Red-1,2-dihexadecanoyl-*sn*-glycero-3-phosphoethanolamine trimethylammonium salt (DHPE), or Uranine, a water-soluble fluorescent compound, GVs were observed in the dispersion without HCl and NaOH under a confocal laser microscope. Under the basic conditions prepared using a 1 M NaOH solution, the GVs were stable in the microscopic field for 24 h. In the dispersion of pH 2 prepared using HCl, no GVs were observed immediately after the observation started, indicating that GVs rapidly collapsed when the HCl solution was added. At pH 3, the GVs gradually shrunk, accompanied by the generation of droplets at the membrane within 30 min (Fig. 2). Similar shrinkage dynamics were observed under an optical microscope for more than 20 GVs with diameters in the range of 4–17 μm in six independent experiments. Shrinkage of GVs with diameters of <2 μm was not distinguishable due to the resolution of the microscope. The vesicle size did not affect the shrinkage dynamics of the observable GVs. Based on previous studies,^30^ it was more appropriate that the GVs were multilamellar rather than unilamellar because the GV dispersions were prepared by the thin film method. Although it is possible that the observed shrinkage dynamics were associated with the lamellarity of the vesicle membrane, estimation of their relationship was difficult because of the limited spatial resolution of the microscope image. A similar shrinkage of GVs forming droplets was also induced by domain formation through the enzymatic hydrolysis of phospholipids to generate diacylglycerols.^31^ Thus, the current observed collapse of GVs could be associated with the compositional change of lipids. In the dispersion containing 10^−3^ M NaCl, the GVs composed of Im hardly shrunk within 30 min. In addition, the GVs were stable at 10^−4^ M HCl. These results clarified that the hydrolysis of Im under stronger acidic conditions affects GV durability, which induces shrinkage. Next, observations of GVs composed of Im and oleic acid at the ratio of 75/25, 50/50, 25/75, and 0/100 (mol%) were carried out under a confocal laser microscope. The total lipid concentration was 1 mM. When the composition of Im/oleic acid was 75/25 or 50/50 (mol%), GVs were observed. In the case of 25/75 and 0/100 (mol%), no GVs were observed. GVs containing 25 mol% oleic acid (Im/oleic acid = 75/25 (mol%)) were tolerant from the basic condition of 10^−3^ M NaOH to an acidic condition of 10^−3^ M HCl. Oleic acid did not form GVs at the tested pH conditions. In addition, when non-reactive *N*-dodecyl-*N*-hexadecyl-*N*,*N*-dimethylammonium bromide (C16–C12) was used instead of Im, size changes and collapse dynamics of GVs were not observed at the tested pH conditions despite the presence and absence of oleic acid (Fig. S1†). Therefore, it was implied that the combination of Im and oleic acid with the appropriate ratio is associated with the durability of GVs over a wide pH range.

### Time course of the Im hydrolysis

**Fig. 3:**
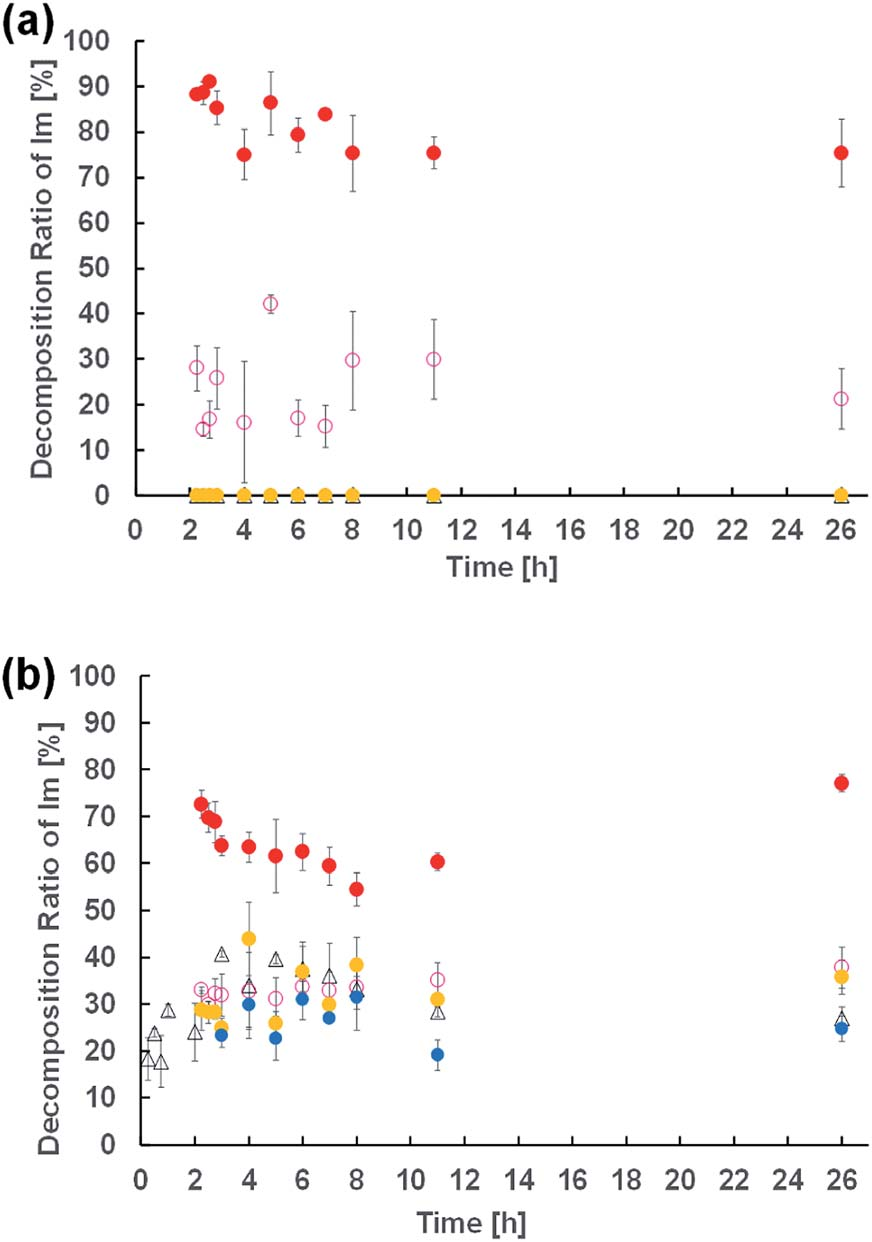
Time course of the hydrolysis ratio of Im in the dispersion of Im (a) and Im/oleic acid (b) calculated by ^1^H NMR. Each measurement was performed in triplicate. Water (black triangle); 10^−2^ M HCl (red filled circle); 10^−3^ M HCl (pink circle); 10^−4^ M HCl (yellow filled circle); 10^−3^ M NaOH (blue filled circle).

**Fig. 4:**
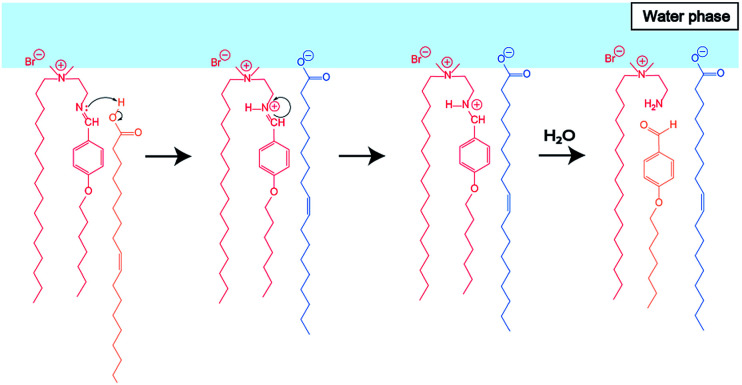
Estimated reaction mechanism of **Im** hydrolysis in the presence of oleic acid during the film swelling process.

To elucidate the effect of changes in lipid composition on the durability of GVs under certain pH conditions, ^1^H NMR analysis was carried out to trace the hydrolysis of Im in D_2_O with/without HCl and NaOH from the film swelling process. Amphiphilic compounds, such as Im and oleic acid, are mainly concentrated in the vesicle membrane rather than monodisperse in the bulk water phase due to their hydrophobicity. Because the Im hydrolysis should occur around the membrane, a higher concentration was used compared to the microscopic observations. The Im hydrolysis was traced integrating the imine proton (*δ* 8.38 ppm) in Im and aldehyde proton (*δ* 9.78 ppm) in HBA after homogenisation by adding CD_3_OD-*d*_4_ containing DMF (*δ* 7.99 ppm) as an internal standard (Fig. S2†). In the absence of oleic acid, the Im hydrolysis hardly progressed in the film swelling process (Fig. 3a). In addition, no significant hydrolysis occurred in the dispersion with/without 10^−4^ M HCl. Under the condition of 10^−3^ M HCl, the reaction proceeded immediately after adding HCl, and the hydrolysis ratio of Im was around 30%. In a more concentrated 10^−2^ M HCl solution, the hydrolysis ratio exceeded 80% within 15 min. Moreover, in the presence of oleic acid, the Im hydrolysis accelerated during the film swelling process, and the hydrolysis ratio reached approximately 25% before the addition of HCl or NaOH (Fig. 3b). This ratio was comparable with the initial composition of oleic acid, suggesting that the imine group of the Lewis base withdrew the proton of oleic acid, and thus the activated imine was rapidly hydrolysed. Indeed, the Im hydrolysis accelerated depending on the amount of oleic acid (Fig. S3†). The reaction mechanism thus indicated the dissociation of oleic acid to generate oleate (Fig. 4). Due to the ion-pairing between the generated anionic oleate and cationic Im and Am in the vesicular membrane, the molecular packing increased. Under conditions of 10^−4^ and 10^−3^ M HCl and 10^−3^ M NaOH, the reaction ratio was almost constant. It was considered that the acidic catalyst did not penetrate into the vesicular membrane because ion-pairing was maintained at low electrolyte concentrations; therefore, no further hydrolysis of Im occurred. However, under the condition of 10^−2^ M HCl, the reaction advanced to about 70% within 15 min. This was probably due to the dissociation of the ion-pairing to regenerate oleic acid because of the 100 times higher concentration of HCl relative to the lipid components. It was therefore concluded that the significant compositional change was related to rapid vesicle shrinkage.

To clarify the influence of the lipid composition on the vesicle durability, observations of dispersions containing 50 mol% hydrolytic products of Im, HBA, and Am were carried out. The size reduction in GVs containing 50 mol% hydrolytes (Im/HBA/Am = 0.5 mM/0.5 mM/0.5 mM) was more frequently observed in 10^−3^ M HCl dispersions when compared to those composed of 1 mM Im for 30 min (Fig. S4†). To analyse the size reduction in GVs, the time until the size stabilized after the addition of HCl (*T*) was measured. As a result of the statistical analysis, *T* of GVs composed of Im (1 mM) was shorter than those of Im/HBA/Am (0.5 mM/0.5 mM/0.5 mM), which was independent of the GV size (Fig. S5†). This result clearly indicated that the lipid composition strongly affected the durability of GVs. In addition, to estimate the relationship between the composition of compounds and GV formation, dispersions without electrolytes were observed (Table S1†). When Im was present, GVs were noticed among the tested compositions. Even though a mixture of HBA and oleic acid (0.75 mM/0.25 mM) afforded droplets, GVs were formed using Am and oleic acid (0.75 mM/0.25 mM, Fig. S6†). At 1 mM Am, no micrometer-scale structures were observed. These results suggest the intermolecular interaction between the cationic compounds, Im and Am, and oleic acid, which contributes to the stronger molecular packing might be an important factor to form stable GVs.

### Estimation of vesicle membrane structure

**Fig. 5:**
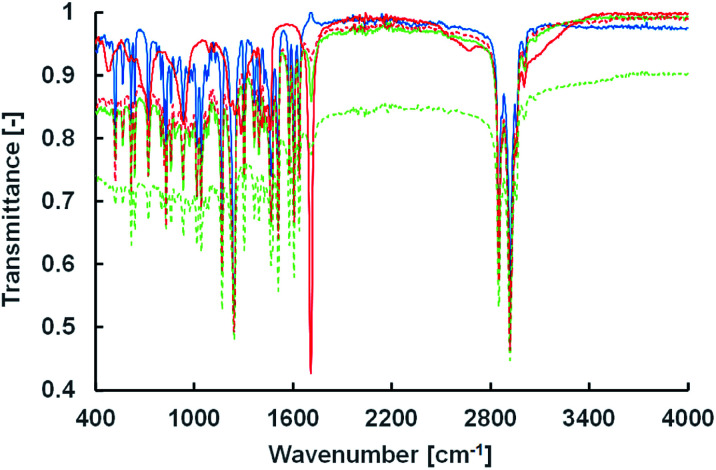
IR spectra of Im/oleic acid = 100/0 (solid blue), 75/25 (solid green), 50/50 (dashed green), 25/75 (dashed red), and 0/100 (solid red) (mol%).

To estimate the vesicle membrane structure based on the lipid composition at the tested pH conditions, two spectroscopic measurements using infrared and fluorescence were carried out. IR measurements were performed using the attenuated total reflection method (Fig. 5). The peak intensities of the carbonyl and hydroxyl groups in oleic acid at 1707 cm^−1^ and 2550–3250 cm^−1^, respectively, weakened in the presence of Im. This suggests an ion–dipole interaction between the quaternary ammonium group in Im and the carbonyl group in oleic acid (and oleate), indicating intermolecular interactions of the cationic compounds, Im and Am, and oleic acid.

**Fig. 6:**
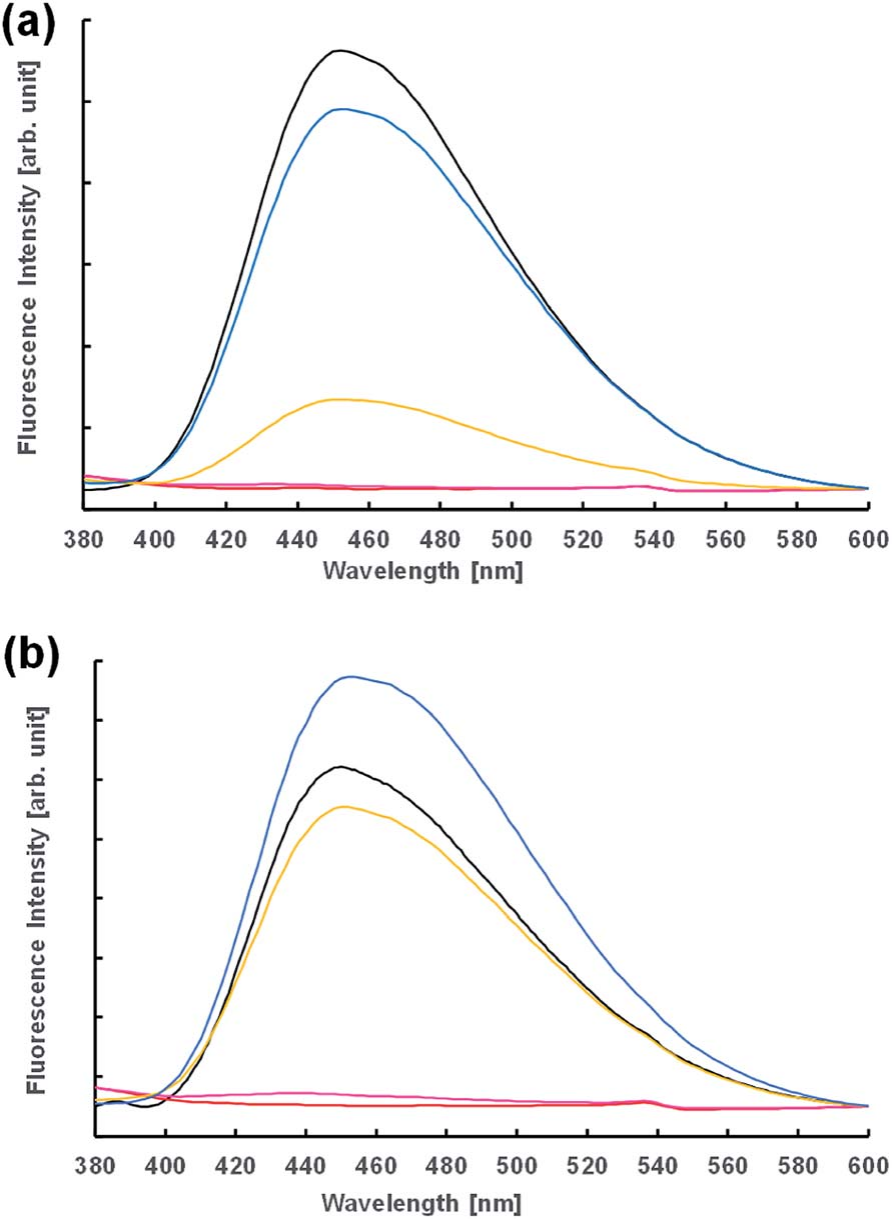
Fluorescence spectra of dispersion with (a) Im and (b) Im/oleic acid = 75/25 (mol%) containing Laurdan. Water (black); 10^−2^ M HCl (red); 10^−3^ M HCl (pink); 10^−4^ M HCl (yellow); 10^−3^ M NaOH (blue).

Next, the vesicular membrane properties were estimated from fluorescence spectra. The environment-responsive fluorescent dye *N*,*N*-dimethyl-6-dodecanoyl-2-naphthylamine (Laurdan) is widely used to investigate the fluidity of vesicle membranes from fluorescence spectra and microscope imaging. It is empirically known that the emission spectrum of Laurdan in a phospholipid membrane depends on the phase state of the membrane.^32,33^ In the dispersion of Im in the presence and absence of 10^−3^ M NaOH 2 h after preparation, the fluorescence spectra were almost the same (Fig. 6). However, the acidic condition of the 10^−4^ M HCl concentration provided a different spectrum; that is, the fluorescence intensity of the dispersion in the presence of oleic acid was significantly higher than that in the absence. This indicated that the membrane structures of vesicles containing oleic acid were harder than those without oleic acid. Moreover, using 10^−3^ M HCl, GVs containing oleic acid were confirmed under a confocal microscope immediately after preparation of the samples (Fig. S7†), but the fluorescence intensity in the spectrum was significantly low (Fig. 6b). Similar spectra were also obtained at pH 2 in the presence and absence of oleic acid. These fluorescence spectra were probably due to the concentration quenching of Laurdan accompanied by a change in the membrane structures. When non-reactive C16–C12 was used instead of Im, no significant differences in the fluorescence spectra were observed depending on the HCl concentration despite the presence and absence of oleic acid (Fig. S8†). Even though the fluorescence spectra using Laurdan did not completely reflect the microscopic observation, these results qualitatively suggested that the membrane structure of GVs composed of Im and oleic acid was more tolerant to a stronger acidic condition.

**Fig. 7:**
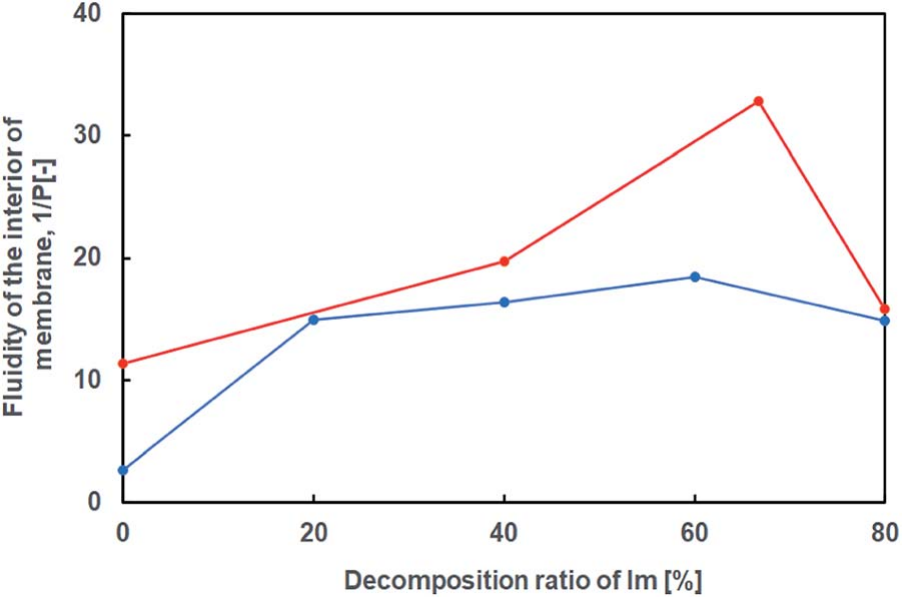
Internal membrane fluidity of Im (blue) and Im/oleic acid (red) vesicles with hydrolysis ratio of Im containing DPH. Im/HBA/Am (blue); Im/HBA/Am/oleic acid (red).

Furthermore, using a hydrophobic compound that exhibits fluorescence in the vesicular membrane, 1,6-diphenyl-1,3,5-hexatriene (DPH), the fluorescence anisotropy in the membrane was estimated with respect to the lipid composition containing the hydrolysates of Im. Since rod-shaped DPH has strong polarisation properties, the fluidity of the lipid bilayer can be estimated from depolarisation in a membrane.^22,34^ The fluidity parameter of the vesicular membrane, 1/*P*, is calculated from *P* = (*I*_‖_ − *GI*_⊥_)/(*I*_‖_ + *GI*_⊥_), *G* = *i*_⊥_/*i*_‖_, where *I*_⊥_, *I*_‖_, *i*_⊥_, and *i*_‖_ are emission intensities for perpendicular and parallel to the vertically and horizontally polarised light, respectively, and *G* is the correction factor. The larger the 1/*P* value, the higher the fluidity of the vesicular membrane. The fluidity of the vesicular membrane was maximised at a composition containing around 60% hydrolysates of Im, and decreased at 80% despite the presence and absence of oleic acid (Fig. 7). This indicated that the membrane structure was not a bilayer but similar to a monolayer^35^ due to the oil component HBA. In the absence of oleic acid, the fluidity increased with a higher composition of hydrolysates. The decrease in fluidity of the membrane containing 80% hydrolysates suggested a drastic change in structures, such as phase separation, that is related to the formation of domains in the membrane.^30^ Since there was a difference in the 1/*P* value in the presence and absence of oleic acid, it was considered that the membrane structure was different in the presence of oleic acid.

**Fig. 8:**
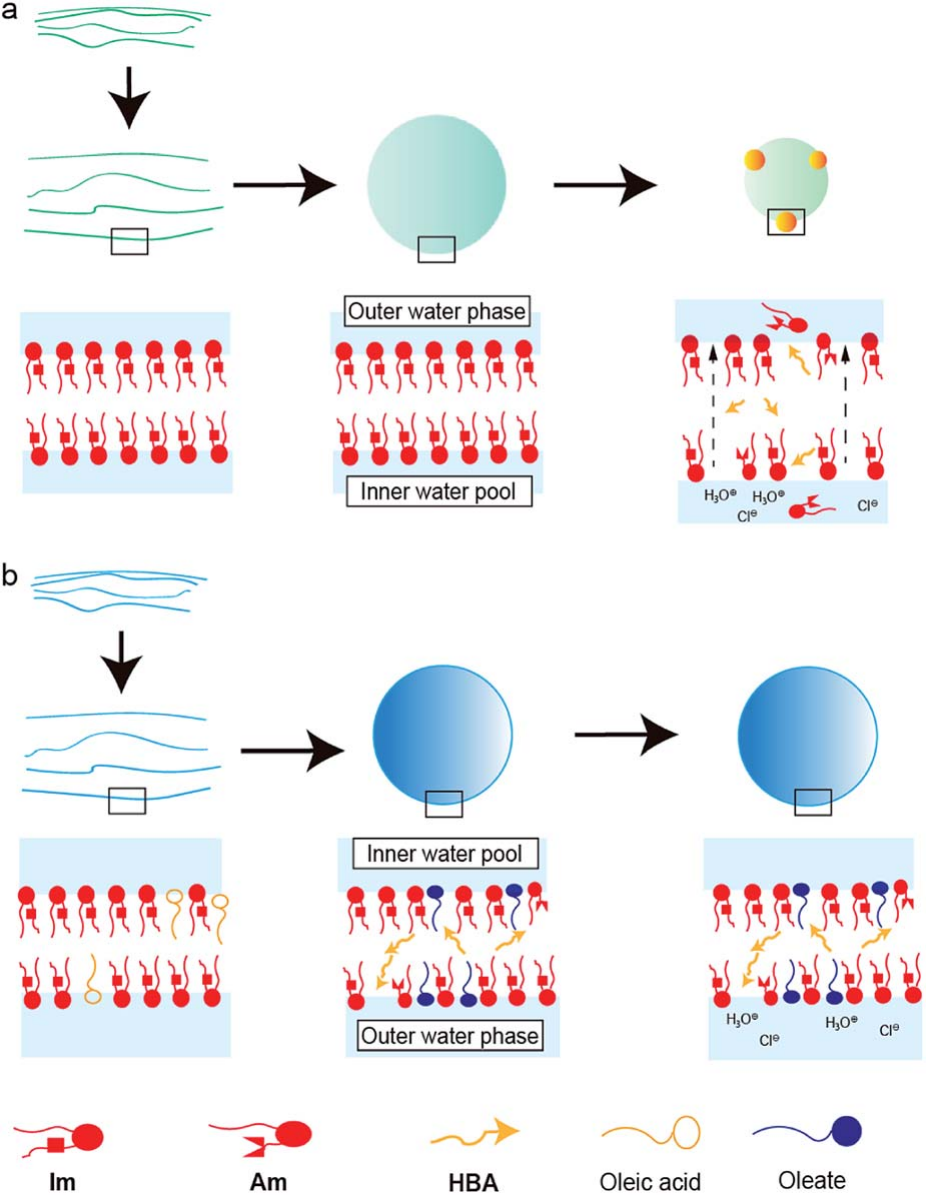
Schematic representations of vesicular membrane structures composed of (a) Im and (b) Im and oleic acid in a dispersion containing 10^−3^ M HCl.

Based on these results, the difference in the durability of Im vesicles with/without oleic acid was interpreted as follows. In the absence of oleic acid, hydrolysis of Im did not proceed during the film swelling process. In addition, to adjust the pH, HCl and NaOH aqueous solutions were added to the dispersion after the vesicle preparation. Thus, the electrolyte concentration in the outer water phase was higher than that in the inner water pool of the GVs. GVs were stable at the low electrolyte concentration of 10^−4^ M HCl, in which hydrolysis of Im did not proceed. Due to the weak molecular packing of the vesicular membrane, Im was rapidly hydrolysed to generate Am and HBA under the more concentrated HCl conditions. In the dispersion containing 10^−3^ M HCl, where Im hydrolysis moderately occurred, the GVs gradually shrank because of the osmotic pressure caused by the difference in the electrolyte concentration between the inner and outer water phases (Fig. 8a). Most Im was hydrolysed in the presence of 10^−2^ M HCl, and thus, the GVs were fragile and rapidly collapsed. Mixing oleic acid with Im GVs generated different vesicular membrane structures (Fig. 8b). During the swelling process of the lipid film, Im hydrolysis proceeded due to the Lewis-basic imine group. This afforded a specific membrane structure with strong molecular packing based on the ion pair between cationic compounds, Im and Am, and anionic oleate (Fig. 4). Even when HCl was added to the GV dispersion at 10^−3^ M, no further hydrolysis of Im occurred; therefore, GVs were tolerant to relatively strong acidic conditions. The molecular system composed of both the imine lipid and oleic acid, where the vesicular membrane structure changes, provides a strategy to construct tolerant vesicles with a wider pH range. In addition, the current model may be linked to the emergence of life. Since the pH in plausible circumstances of early earth is estimated from 2 to 11,^36,37^ the durability of structures under a wider pH would have advantages for selection. The imine bond is one of the dynamic covalent bonds that can be hydrolysed and generated under mild conditions and has drawn attention to construct prebiotic supramolecular self-assemblies.^38,39^ Oleic acid/oleate also represents a good model system for the evolution of life because fatty acids are considered prebiotic molecules.^40^ Therefore, it is considered that the designed molecular system affords one possibility for the chemical evolution of protocells that have tolerance to a wider pH range.

## Conclusions

In this study, pH-responsive GVs were investigated using a mixture of cationic lipids having an imine bond and oleic acid. From the ^1^H NMR, IR, and fluorescence spectra, it was revealed that the durability of GVs containing oleic acid under a wide pH range from basic to acidic conditions was related to the strong molecular packing, which was based on the formation of ion-pairing between cationic lipids and oleate. This enhancement of membrane structures occurred by hydrolysis of the imine lipid to promote dissociation of oleic acid during the film swelling process. GVs were fragile and rapidly collapsed under more acidic conditions due to dissociation of the ion-pairing followed by further hydrolysis of the imine lipid caused by the ion strength. The current molecular system can not only provide the methodology to generate tolerant vesicles for a wide pH range from the viewpoint of supramolecular chemistry but also show a possibility in the chemical evolution of protocells, especially selection.

## Conflicts of interest

The authors declare no conflict of interest.

## Supplementary Material

RA-010-D0RA06822E-s001
