# Supplementary material for: pH-Tolerant giant vesicles composed of cationic lipids with imine linkages and oleic acids
Source: RSC Adv. 2020 Sep 15;10(56):34247–53. doi: 10.1039/d0ra06822e (PMC9056790; doi:10.1039/d0ra06822e)
Supplement: RA-010-D0RA06822E-s001 [file RA-010-D0RA06822E-s001.pdf]

Electronic Supplementary Material (ESI) for RSC Advances.
This journal is © The Royal Society of Chemistry 2020

pH-Tolerant giant vesicles composed of cationic lipids with imine linkages and oleic acids

Daichi Sawada, Ayana Hirono, Kouichi Asakura, Taisuke Banno

Department of Applied Chemistry, Faculty of Science and Technology, Keio University, 3-14-1 Hiyoshi, Kohoku-ku, Yokohama 223-8522, JAPAN

Supplementary information index

**Synthetic procedures**	**p. 2**
**Figures**	**p. 5**
**Table**	**p. 10**
**References**	**p. 10**

Synthetic procedures

Synthesis of the amphiphile having an imine linkage (Im)

Im was synthesized according to the procedure given in Scheme S1.

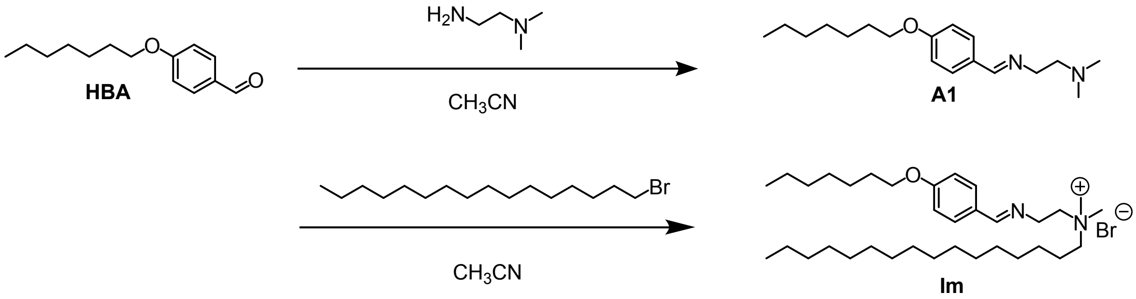

Scheme S1. Synthesis of **Im** having a hydrolysable group.

*Synthesis of (E)-2-((4-(heptyloxy)benzylidene)amino)ethylene-N,N-dimethylamine(**A1**)*

n-Heptyloxybenzaldehyde (**HBA**) (2.20 g, 10 mmol) and *N,N*-dimethylethylenediamine (1.32 g, 15 mmol) were added to MeCN (20 mL). A catalytic amount of acetic acid (1 drop) was added, and then the mixture was stirred at 80 °C for 12 h. After the reaction, the solvent was removed by evaporation under reduced pressure. The obtained crude product dissolved in 15 mL of ethyl acetate was washed three times with a 5% aqueous $NaHCO_3$ solution, and then dried over anhydrous magnesium sulfate. The solvent of filtrate was evaporated under reduced pressure to obtain the mixture of **A1** and **HBA** (2.84 g), as yellow viscous liquid, whose ratio was 1/0.07 (mol/mol) analyzed by ^{1}H NMR spectrum using $CDCl_3$.

*Synthesis of N-(E)-((2-((4-(heptyloxy)benzylidene)amino)ethyl)-N-hexadecyl-N,N-dimethylammonoum bromide (**Im**)*

A mixture of **A1** including **HBA** (2.84 g) and 1-bromohexadecane (2.68 g, 7.76 mmol) was reacted in anhydrous MeCN (20 mL) with stirring at 70 °C for 24 h. After the reaction, the solvent was removed by evaporation under reduced pressure. The crude product was dissolved in chloroform (10 mL) at 25 °C, and then ethyl acetate (50 mL) was added at room temperature to reprecipitate to obtain **Im** (3.36 g), as a white crystal, in a yield of 56% in a two-step reaction.

^{1}H-NMR (400 MHz, $CDCl_3$) δ 8.31 (s, 1H), 7.61 (d, J = 8.9 Hz, 2H), 6.90 (d, J = 8.7 Hz, 2H), 4.14–3.89 (m, 6H), 3.74–3.58 (m, 2H), 3.58–3.40 (m, 6H), 1.86–1.69 (m, 4H), 1.50–1.06 (m, 36H), 0.90–0.85 (m, 6H).

^{13}C NMR (125 MHz, $CDCl_3$); δ 163.9, 162.1, 130.1, 127.9, 114.8, 68.3, 65.8, 64.0, 55.0, 52.3, 32.0, 31.9, 29.8, 29.7, 29.5, 29.2, 22.8, 22.7, 14.2, 14.2.

HR-ESI-MS (*m*/*z*): calcd. for $C_{34}H_{62}N_2OBr$ 514.4857 $[M–Br]^+$; found 515.4835 $[M–Br]^+$.

Synthesis of the amphiphile having an amine group (Am) [1]

Am was synthesized according to the procedure of Scheme S2 reported by Chen et al[1].

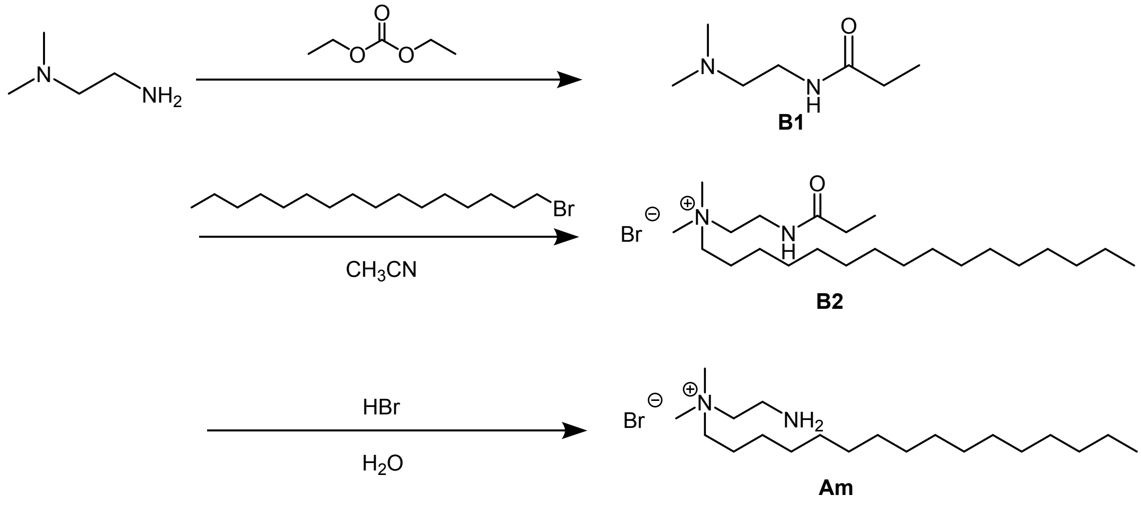

Scheme S2. Synthesis of **Am** having amine linkage.

*Synthesis of N-(2-(dimethylamino)ethyl)propionamide (**B1**)*

N, N-dimethyl-*N*-2-ethylamine (4.40 g, 0.05 mol) and diethyl carbonate (7.10 g, 0.06 mol) were reacted at 70 °C. for 48 h. After the reaction, the unreacted materials were removed by evaporation under reduced pressure to obtain **B1** (7.20 g) as brownish oil, in a yield of 89%.

^{1}H-NMR (400 MHz, $CDCl_3$) δ 5.20 (s, 1H), 4.10 (q, 2H), 3.25 (t, J = 5.4 Hz, 2H), 2.40 (t, J = 5.8 Hz, 2H), 2.23 (s, 6H), 1.23 (t, J = 7.1 Hz, 3H).

*Synthesis of N-(2-propionamidoethyl)hexadecyl-N,N-dimethylammoinium bromide (**B2**)*

A mixture of **B1** (100 mg, 0.618 mmol) and 1-bromohexadecane (193 mg, 0.633 mmol), was reacted in acetonitrile (2 mL) at 70 °C for 48 h. After the reaction, the solvent was removed by evaporation under reduced pressure. The crude product was obtained by recrystallization using acetonitrile/diethyl ether (1:1, v/v, 5.0 mL). Thereafter, the crystals were washed with hexane to obtain **B2** (92.6 mg), as a white crystal, in a yield of 33%.

^{1}H-NMR (400 MHz, $CDCl_3$) δ 6.74 (s, 1H), 4.09 (q, J = 7.1 Hz, 2H), 3.80 (s, 4H), 3.59–3.48 (m, 2H), 3.39 (s, 6H), 1.71 (br, 2H), 1.34–1.21 (m, 32H), 0.87 (t, J = 6.8 Hz, 3H).

HR-ESI-MS (*m*/*z*): calcd. for $C_{23}H_{49}N_2OBr$ 385.3789 $[M–Br]^+$;found 385.3690 $[M–Br]^+$.

*Synthesis of N-(2-aminoethyl)-N-hexadecyl-N,N-dimethylammoinium bromide(**Am**)*

A mixture of **B2** (29.4 mg, 0.0629 mmol) and 47% aqueous HBr solution (500 μL) was reacted in a screw-capped tube at 100 ° C for 3 days. After the reaction, the solvent was removed by evaporation under reduced pressure. Purification was carried out by recrystallization using ethanol/diethyl ether

(1/1, v /v, 1.0 mL) to obtain **Am** (25. 3mg), as a brown crystal, in a yield of 98%.

^{1}H-NMR (400 MHz, D_2O) *δ* 3.74 (d, *J* = 8.2 Hz, 2H), 3.62–3.48 (m, 4H), 3.24 (s, 6H), 1.82 (br, 2H), 1.38–1.28 (m, 26H), 0.86 (t, *J* = 6.6 Hz, 3H).

HR-ESI-MS (*m*/*z*): calcd. for $C_{20}H_{45}N_2Br$ 313.3577 $[M–Br]^+$; found 313.3463 $[M–Br]^+$.

Figures

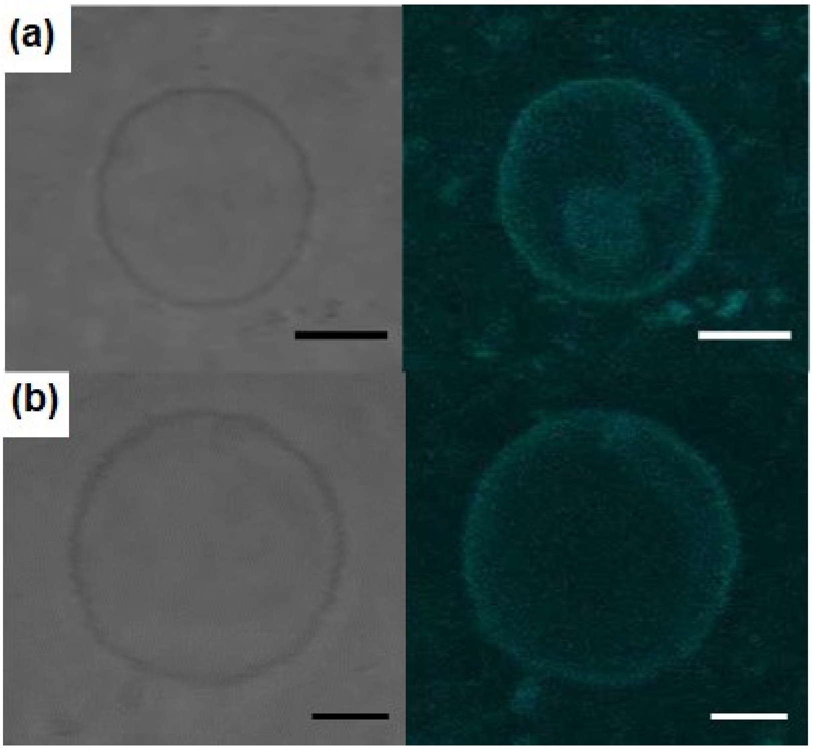

Fig. S1 Typical confocal laser microscope images of GVs composed of (a) **C16-C12** and (b) **C16-C12**/oleic acid = 75/25 (mol%) containing 1 mM Laurdan in 10^{-3} M HCl dispersion. Scale bar: 10 μm.

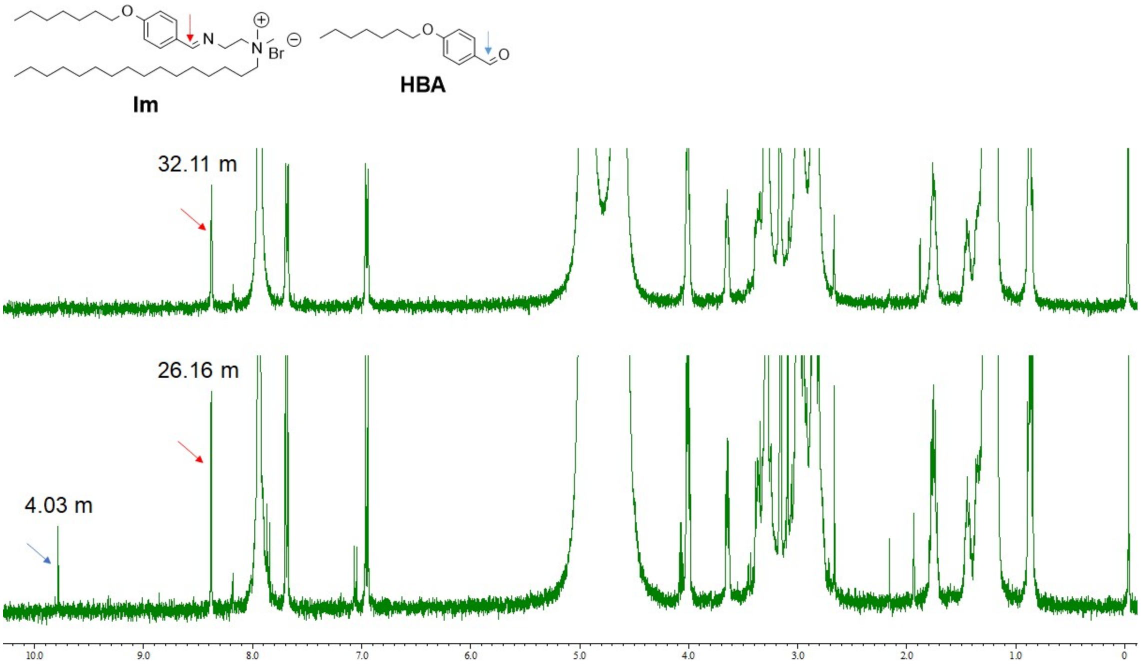

Fig. S2 ^{1}H NMR spectra of D_2O and CD_3OD-d_4 (100 mL/650 mL) mixed solution containing **Im** (7.5 mM), HCl (10 mM), and DMF (36.5 mM), used as a standard compound 0 min (top) and 30 min (bottom) after addition of the concentrated 1 M HCl solution.

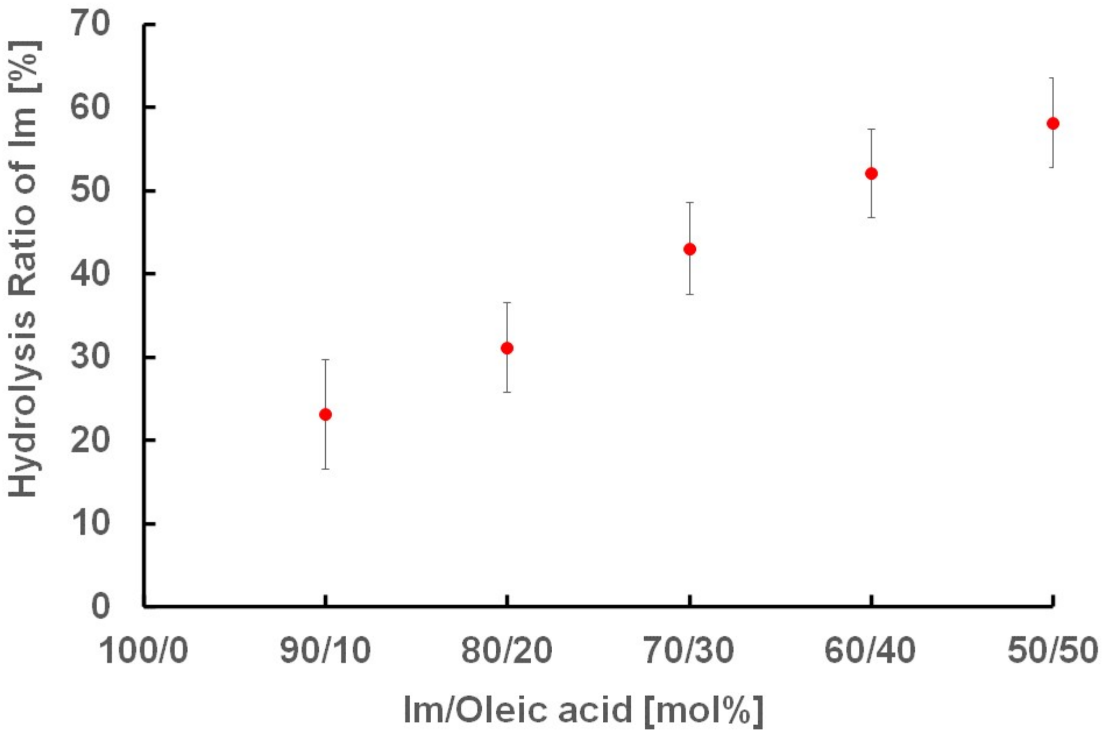

Fig. S3 Hydrolysis ratio of **Im** in the dispersion of **Im**/oleic acid calculated by ^{1}H NMR after the swelling process in D_2O for 2 h. Each measurement was performed three times.

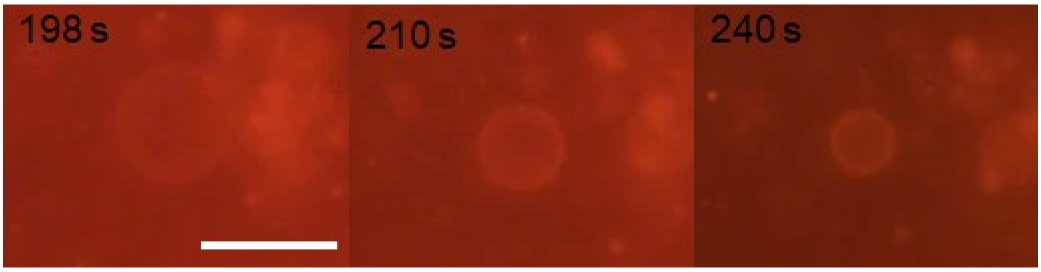

Fig. S4 Typical microscope images of GVs composed of **Im**/**Am**/**HBA** = 0.5 mM/0.5 mM/0.5 mM in 10^{-3} M HCl dispersion. Scale bar: 20 µm.

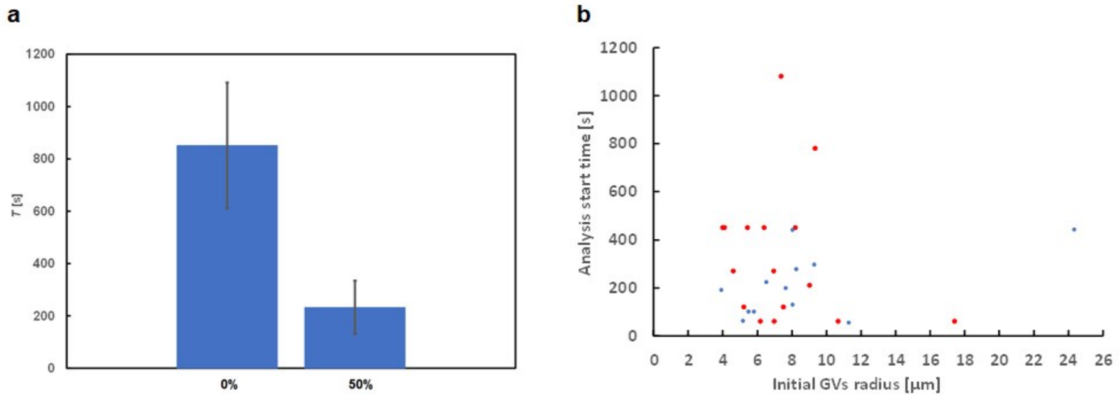

Fig. S5 (a) Time until the size became almost constant after addition of HCl at a concentration of 10^{-3} M (T) of GVs composed of 1 mM **Im** (0%) and **Im**/**Am**/**HBA** = 0.50 mM/0.5 mM/0.5 mM (50%) ($n \geq 10$). Calculation of the f-test was confirmed that 0% and 50% was an unequal variance because the p value was <0.05. From the calculation of the t-test, it was also confirmed that T was significant because the p value was <0.05. (b) T of 10 or more GVs composed of 1 mM **Im** (red) and **Im**/**Am**/**HBA** = 0.50 mM/0.5 mM/0.5 mM (blue) ($n \geq 10$). There was no difference between 0% and 50% depending on the initial size. From these, the statistical difference in GVs radius changes in the difference lipid composition was more clearly confirmed.

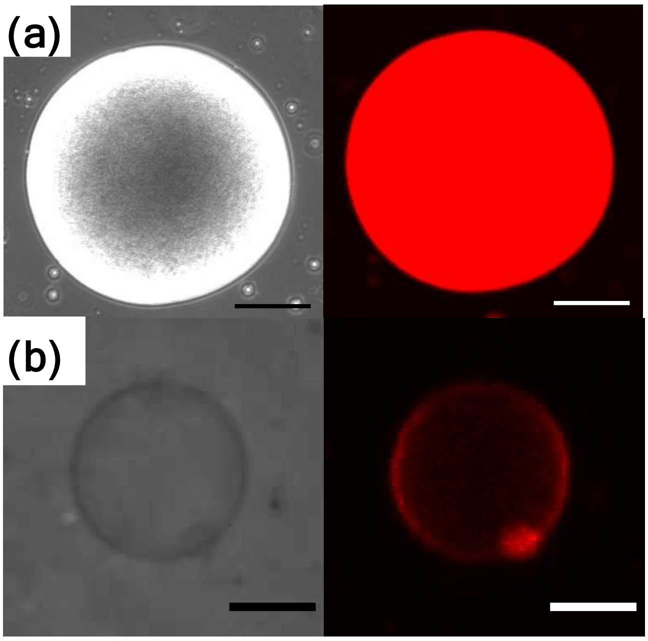

Fig. S6 Typical confocal laser microscope bright field (left) and fluorescence (right) images of aggregation composed of (a) **HBA**/oleic acid = 75/25 (mol%) and (b) **Am**/oleic acid = 75/25 (mol%) and Texas Red-DHPE 1 μM dispersion. Scale bar: 10 μm.

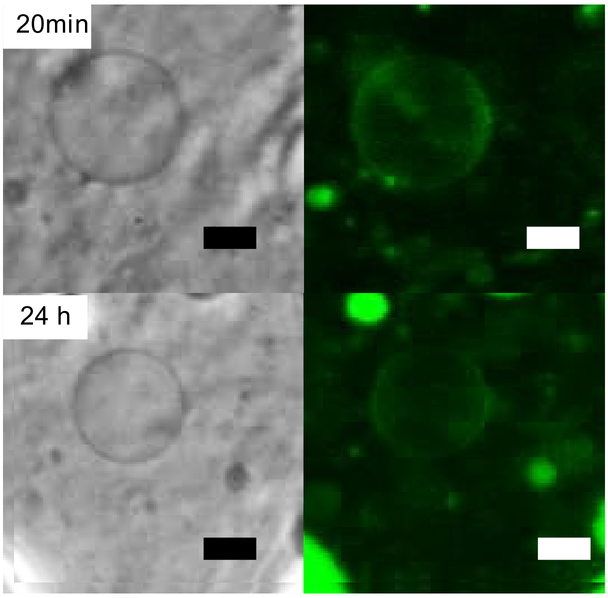

Fig. S7 Confocal microscope images of dispersion containing **Im** and oleic acid (75/25 mol%) at pH 3. Scale bar: 10 μm.

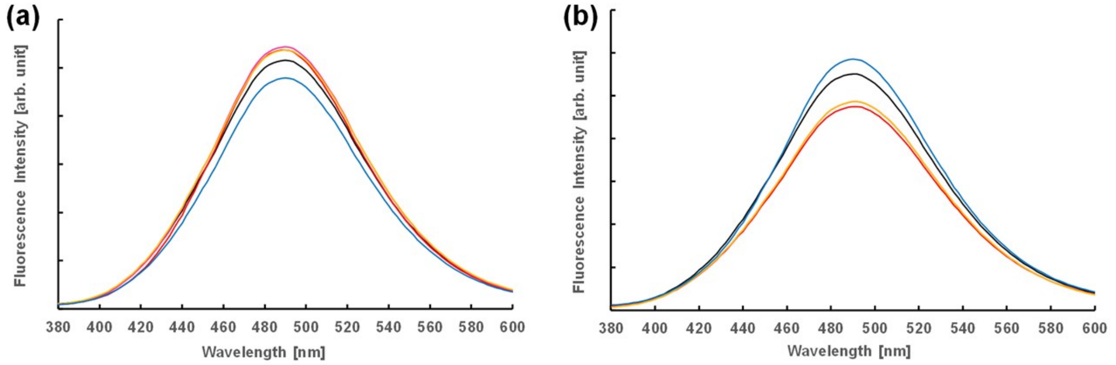

Fig. S8 Fluorescence spectra of dispersion containing (a) **C16-C12** and (b) **C16-C12**/oleic acid = 75/25 (mol%) containing Laurdan. Water (black); 10^{-2} M HCl (red); 10^{-3} M HCl (pink); 10^{-4} M HCl (yellow); 10^{-3} M NaOH (blue).

Table

Table S1. Microscopic observation of samples composed of **Im**, **HBA**, **Am**, and oleic acid.

**Im** [mM]	**Am** [mM]	**HBA** [mM]	Oleic acid [mM]	Observation results
0.700	0	0.300	0	GVs
0.400	0	0.600	0	GVs
0.525	0	0.225	0.250	GVs
0.300	0	0.450	0.250	GVs
0	0	0.750	0.250	oil droplets
0.700	0.300	0	0	GVs
0.400	0.600	0	0	GVs
0.525	0.225	0	0.250	GVs
0.300	0.450	0	0.250	GVs
0	0.750	0	0.250	GVs
0	1.000	0	0	n.o.*

* Any micrometer-sized aggregates were not observed.

References

1 Ying Chen, Heng Yang, Weijun Tang, Xinhui Cui, WeiWang, Xiangyu Chen, Yuan Yuan and Aiguo Hu, *J. Mater. Chem. B*, 2013, **1**, 5443-5449.
